# Receptor selectivity between the G proteins Gα_12_ and Gα_13_ is defined by a single leucine-to-isoleucine variation

**DOI:** 10.1096/fj.201801956R

**Published:** 2019-01-02

**Authors:** Amanda E. Mackenzie, Tezz Quon, Li-Chiung Lin, Alexander S. Hauser, Laura Jenkins, Asuka Inoue, Andrew B. Tobin, David E. Gloriam, Brian D. Hudson, Graeme Milligan

**Affiliations:** * Centre for Translational Pharmacology, Institute of Molecular, Cell, and Systems Biology, College of Medical, Veterinary, and Life Sciences, University of Glasgow, Glasgow, United Kingdom;; † Department of Drug Design and Pharmacology, University of Copenhagen, Copenhagen, Denmark; and; ‡ Graduate School of Pharmaceutical Sciences, Tohoku University, Sendai, Japan

**Keywords:** GPCR, GPR35, G protein barcode, genome editing

## Abstract

Despite recent advances in structural definition of GPCR–G protein complexes, the basis of receptor selectivity between G proteins remains unclear. The Gα_12_ and Gα_13_ subtypes together form the least studied group of heterotrimeric G proteins. G protein–coupled receptor 35 (GPR35) has been suggested to couple efficiently to Gα_13_ but weakly to Gα_12_. Using combinations of cells genome-edited to not express G proteins and bioluminescence resonance energy transfer–based sensors, we confirmed marked selectivity of GPR35 for Gα_13_. Incorporating Gα_12_/Gα_13_ chimeras and individual residue swap mutations into these sensors defined that selectivity between Gα_13_ and Gα_12_ was imbued largely by a single leucine-to-isoleucine variation at position G.H5.23. Indeed, leucine could not be substituted by other amino acids in Gα_13_ without almost complete loss of GPR35 coupling. The critical importance of leucine at G.H5.23 for GPR35–G protein interaction was further demonstrated by introduction of this leucine into Gα_q_, resulting in the gain of coupling to GPR35. These studies demonstrate that Gα_13_ is markedly the most effective G protein for interaction with GPR35 and that selection between Gα_13_ and Gα_12_ is dictated largely by a single conservative amino acid variation.—Mackenzie, A. E., Quon, T., Lin, L.-C., Hauser, A. S., Jenkins, L., Inoue, A., Tobin, A. B., Gloriam, D. E., Hudson, B. D., Milligan, G. Receptor selectivity between the G proteins Gα_12_ and Gα_13_ is defined by a single leucine-to-isoleucine variation.

Recent years have seen enormous advances in knowledge of the structural organization of members of the GPCR superfamily (1–3), with many examples now available of inactive state structures of rhodopsin-like, class A GPCRs. Moreover, although less abundant, a significant number of active state or active state–like structures have also been described (4). A common distinction between inactive and active states is the reorientation of the intracellular facing elements of the transmembrane helices, particularly of transmembrane domain VI (5). It has long been appreciated that the C-terminal α5 helix of G protein α subunits engages with the active state receptor and that this is a crucial step toward triggering guanine nucleotide exchange on the G protein α subunit to initiate steps that result in regulation of signal transduction cascades (6, 7). Although still limited in number (8–11), structural complexes of an active state GPCR and a G protein have provided validation of such models that were developed over many years. Such a role of the extreme C-terminal region of the G protein α subunit in engaging the agonist-occupied receptor had been predicted. For example, the site of pertussis toxin–catalyzed ADP-ribosylation, which prevents effective interactions between GPCRs and Gα_i_-family G proteins, is the Cys residue located 4 aa from the C-terminal tail in each of these Gα subunits (12), whereas the molecular basis of the uncoupled mutation of Gα_s_ that also prevents interaction with appropriate GPCRs is a single amino acid alteration 6 residues from the C-terminal tail (12). Moreover, the capacity to produce chimeric G proteins that alter GPCR interaction selectivity by providing as few as the C-terminal 5–10 aa of a G protein α subunit have been integral to such understanding (12).

Partially for convenience of classification, GPCRs are often designated as being coupled primarily to members of one of the 4 families, Gα_s_, Gα_i_, Gα_q_/Gα_11_, and Gα_12_/Gα_13_, of heterotrimeric G proteins. In many cases, this is well justified because the receptor in question clearly signals predominantly in a manner consistent with engagement with members of only one of the 4 groups. However, in many other cases studies using *in vitr*o expression systems indicate a broader G protein–coupling profile with, in certain examples, a degree of interaction with virtually all G proteins tested (13). More importantly, a capacity to activate G proteins from more than 1 G protein family is also true for many receptors expressed natively, and this may dictate distinct physiologic outcomes. For example, although free fatty acid receptor 4 promotes secretion of the incretin glucagon-like peptide 1 by activation of pertussis toxin–insensitive G proteins of the Gα_q/11_ family (14), regulation of release of the satiety hormone ghrelin requires activation of one or more pertussis toxin–sensitive Gα_i_-family G protein (15). Equally, although free fatty acid receptor 2 acts to counter lipolysis in mouse white adipose tissue *via* a pertussis toxin–sensitive mechanism (16), effects of this receptor on secretion of glucagon-like peptide 1 are instead mediated by Gα_q/11_-family G proteins (16). Although such examples are clearly defined, there is often less understanding of the importance of selective interactions of a receptor with different members from within one of the G protein families and little insight into the molecular basis of such selectivity, and there are currently no comparative structures of a single GPCR in complex with 2 different G proteins.

Although clearly involved in GPCR-mediated cytoskeletal organization or reorganization and the consequences thereof, the least studied of the Gα protein family is the 2-member Gα_12_/Gα_13_ subgroup (17, 18). Although it is activated by many GPCRs, relatively little has been published on these because of a lack of selective inhibitors, and assays to measure their activation are challenging (19). However, although Gα_12_ and Gα_13_ are generally coexpressed, mouse knock-out studies demonstrate that they are not interchangeable (17), and certain GPCRs appear to couple selectively to Gα_12_, Gα_13_, or both. An interesting case in point is G protein–coupled receptor 35 (GPR35) (20). Although officially an orphan receptor, in that suggestions of its endogenous activator or activators remain controversial (20–24), a wide range of surrogate agonists are available (20, 25). Although coupling to Gα_12_/Gα_13_ is well established, the basis of potential selectivity in so doing is not. Herein we address 2 key questions: Does GPR35 selectively activate Gα_13_ over Gα_12_, and, if so, what is the molecular basis of this difference?

## MATERIALS AND METHODS

Materials for cell culture were from MilliporeSigma (Burlington, MA, USA) or Thermo Fisher Scientific (Waltham, MA, USA). Polyethylenimine linear MW-25000 was from Polysciences (Warrington, PA, USA). Zaprinast, lodoxamide, pamoic acid, and a specific GPR35 antagonist (CID2745687) were purchased from commercial sources. Compound 1 {4-[(*Z*)-[(2*Z*)-2-(2-fluorobenzylidene)-4-oxo-1,3-thiazolidin-5-ylidene]methyl]benzoic acid} is described in Neetoo-Isseljee *et al.* (25). Bufrolin [5,6-dimethyl-2-nitro-1*H*-indene-1,3(2*H*)-dione] is described in Mackenzie *et al.* (26). 6-bromo-8-(4-methoxybenzamido)-4-oxo-4H-chromene-2-carboxylic acid (PSB-13253) (27) was a gift from Christa Muller and Dominik Thimm (University of Bonn, Bonn, Germany). In all cases the GPR35a splice variant of human GPR35 (hGPR35) was used.

### Generation of bioluminescence resonance energy transfer systematic protein affinity strength modulation sensors

Systematic protein affinity strength modulation (SPASM) sensors consisting of the receptor of interest [hGPR35a or mouse GPR35 (mGPR35)] were generated based on previously reported Förster resonance energy transfer SPASM sensors (28). These sensors consist of a single construct of receptor, fused at its C terminus to mCitrine, followed by an ER/K α helical linker (29), the bioluminescent protein Nanoluc (Promega, Madison, WI, USA), and to a peptide corresponding to the final 27 aa of Gα_12_, Gα_13_, or Gα_q_. To ensure flexibility within the sensor unit, (GlySerGly)_4_ linkers were included to separate each element—mCitrine, ER/K linker, Nanoluc, G peptide—of the sensor. Sensors were cloned using PCR and a seamless-end homology cloning approach (Thermo Fisher Scientific), generating constructs that did not contain restriction enzyme sites separating the various sensor elements. All constructs were fully sequenced prior to use.

### Cell culture

Clones of cells genome-edited to lack expression of Gα_q_/Gα_11_, Gα_12_/Gα_13_ or each of Gα_q_/Gα_11_/Gα_12_/Gα_13_ were derived from parental human embryonic kidney 293 (HEK293) cells as previously described (30). Along with HEK293-T (T antigen) cells and Flp-In T-REx 293 cells harboring various sensor constructs these were grown in DMEM (MilliporeSigma) supplemented with 10% fetal bovine serum, 2 mM l-glutamine, and 1% penicillin/streptomycin. Cells were incubated in a humidified CO_2_ incubator at 37°C.

### Bioluminescence resonance energy transfer studies using SPASM sensors

Flp-In T-REx 293 cell lines stably harboring the SPASM sensor of interest were seeded into poly-d-lysine coated Greiner white 96-well plates. Doxycycline (100 ng/ml) was added 3–4 h after seeding to induce expression of the sensor, and cells were incubated overnight at 37°C in 5% CO_2_. For transiently expressed constructs, HEK293-T cells were seeded to obtain 60% confluence the following day. The cells were then transfected with 30 ng DNA and 180 ng polyethylenimine per well 2 d prior to the experiment. Thirty minutes before the assay, cells were washed with HBSS buffer containing 10 mM HEPES and incubated in the same buffer at 37°C. Because bioluminescence resonance energy transfer (BRET) provides a ratiometric signal and herein the constructs are single polypeptides, outcomes are anticipated to be independent of expression levels.

### Kinetic studies

Coelenterazine-h was added to each well to give a final concentration of 5 μM 15 min prior to the read. Each well was read for 30 s before addition of a ligand and read for a further 90 s before any second addition. Wells were read throughout a 6-min window using a PheraStar plate reader (BMG Labtech, Cary, NC, USA) for 0.5-s simulations with filters for wavelengths of 475 nm for Nanoluc luminescence and 535 nm for mCitrine emission. The raw value for 535 nm was divided by the 450-nm value to obtain a BRET ratio. This value was then divided by the baseline value of the first 30 s of read before the addition of any ligand to obtain a fold change over baseline. This value for the vehicle-only additions for each cell type was subsequently subtracted from the corresponding treatment to give the final value used for analysis.

### Endpoint assay

Coelenterazine-h was added to each well to give a final concentration of 2.5 μM 5 min prior to the addition of ligands. Wells were read 5 min after addition of ligands and hence 10 min after addition of coelenterazine-h. Each well was read on a ClarioStar plate reader for 0.5 s consecutively at wavelengths of 450 ± 45 nm for the Nanoluc luminescence and 550 ± 45 nm for mCitrine emission. The raw value for 550 nm was divided by the 450 nm value to obtain a BRET ratio. This value was subsequently divided by the value of the vehicle-only addition to obtain a final fold change over baseline value.

### BRET studies using full-length G protein α subunits

The Nanoluc luciferase coding sequence was inserted in the human Gα_13_ sequence immediately following residue Arg128, with (GlySerGly)_4_ linkers on either side. This position has previously been verified as maintaining Gα_13_ function in Förster resonance energy transfer studies that introduced a fluorophore at this location (31). HEK293 cells genome-edited to lack expression of both Gα_12_ and Gα_13_ were transiently transfected 2 d prior to the experiment to coexpress hGPR35 tagged at the C terminus with enhanced yellow fluorescent protein (eYFP) and the indicated Nanoluc-containing Gα_13_ protein. BRET assays were conducted as described above. Subsequent studies employed this base Nanoluc-containing Gα_13_ construct in which residues G.H5.22 (Gln) and G.H5.23 (Leu) were altered to the corresponding residues of Gα_12_ (G.H5.22, Asp) or (G.H5.23, Ile).

### β-Arrestin-2 interaction studies

These employed a BRET-based assay. HEK293-T cells were transfected transiently to coexpress hGPR35-eYFP and β-arrestin-2 tagged with *Renilla* luciferase (32). Agonist-induced proximity between these 2 proteins generated enhanced BRET signal upon addition of the luciferase substrate coelenterazine-h.

### High content imaging/internalization studies

These were performed essentially as described by Mackenzie *et al.* (26). Briefly, Flp-In T-REx 293 cells harboring the hGPR35-SPASM sensor of interest were seeded into poly(d-lysine)–coated black clear-bottom 96-well plates at a density of 80,000 cells per well. Receptor expression was induced *via* the addition of doxycycline (100 ng/ml) 6 h after seeding. Twenty-four hours later, cells were washed twice with serum-free medium and incubated with ligand for 45 min at 37°C, before being fixed with paraformaldehyde (4% v/v). Cells were washed with PBS and incubated with 10 μg/ml Hoechst nuclear stain at 37°C for 30 min to allow determination of cell number. Receptor internalization was quantified using an ArrayScan II high content imager (Thermo Fisher Scientific), which detected the location of the mCitrine fluorescent protein component of the sensor as it was trafficked to endocytic recycling compartments.

### TGF-α shedding assay

TGF-α shedding in response to activation of hGPR35 was assessed as previously described (30, 33). Briefly, parental HEK293 cells or cells of clones genome-edited to lack expression of various G protein α subunit combinations (30, 34, 35) were seeded onto 6-well plates (5 × 10^5^ cells per well) 24 h prior to transfection. Cells were transfected with 0.5 μg of a plasmid containing an alkaline phosphatase (AP) fusion protein of TGF-α, AP-TGF-α, and 0.1 μg hGPR35. The next day, transfected cells were trypsinized, washed with HBSS, and reseeded on to 96-well plates 30 min prior to treatment with ligands. Cells were then treated with agonists and incubated at 37°C for 1 h. After such treatment, conditioned medium was transferred into other 96-well plates. A solution of 10 mM paranitrophenylphosphate, 40 mM Tris-HCl (pH 9.5), 40 mM NaCl, and 10 mM MgCl_2_ was added to both cell and conditioned medium plates, and the absorbance was measured at 405 nm before and after a 60-min incubation at 25°C. TGF-α shedding was calculated as the percentage increase of optical density at 405 nm in conditioned medium plate/overall total increase of optical density at 405 nm (from both conditioned medium and cell plates).

### Modeling studies

We first investigated sequence-based selectivity determinants in Gα_13_ by evolutionary conservation for G.H5.23 with its orthologs and paralogs as previously described in Flock *et al.* (36). Structure-based sequence alignments were performed using the GPCR Database numbering for GPCRs and the Common Gα Numbering scheme for G proteins (36, 37). In this scheme, G is the G protein, H5 is the α5 helix, and the numerical value is the position from the start of that helix (*e.g.*, residue 23), hence G.H5.23. Structural inspections were performed on the receptor-G protein complexes of β_2_-adrenoceptor-Gα_s_ (Protein Data Bank: 3SN6) and µ-opioid receptor-Gα_i1_ structure (Protein Data Bank: 6DDE). Structures were prepared using the protein preparation wizard in Maestro (Schrödinger, New York, NY, USA) using default settings and including addition of missing residues followed by H-bond assignment. Interface contacts between G.H5.23 and the receptor (including backbone interactions) were calculated using Arpeggio using default settings (maximum range of interaction set to 5.0 Å) (38). Superpositioning, rotation calculations, and visualizations were performed using PyMol (PyMol Molecular Graphics System, v.2.0; Schrödinger).

## RESULTS

### hGPR35 can interact with G_12_/G_13_ but not with G_q_/G_11_

**Figure 1:**
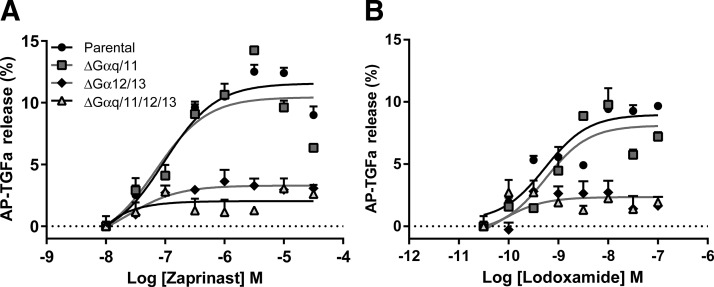
hGPR35 promotes shedding of an AP-tagged form of TGF-α *via* activation of G_12_/_13_ but not *via* G_q_/G_11._ The ability of varying concentrations of either zaprinast (*A*) or lodoxamide (*B*) to promote shedding of an AP-tagged form of TGF-α was assessed following cotransfection of hGPR35 and AP-TGF-α into each of parental HEK293 cells (circles) or clones of HEK293 cells that had been genome-edited to lack expression of Gα_12_ + Gα_13_ (diamonds), Gα_q_ + Gα_11_ (squares), or a combination of Gα_q_, Gα_11_, Gα_12_, and Gα_13_ (triangles). Basal levels of AP-TGF-α release were subtracted. Data represent means ± sd in triplicates from each group of a single experiment representative of 3 performed.

To assess the ability of hGPR35 to interact productively with G proteins of the Gα_12_/Gα_13_ and Gα_q_/Gα_11_ families, we employed HEK293 cells, because they express all 4 of these G proteins (30), and a TGF-α shedding assay (30, 33) because it has previously been established that the TGF-α shedding endpoint is promoted by receptors that stimulate activation of any combination of G_q_, G_11_, G_12_, and G_13_ (30). Following transient cointroduction of hGPR35 and an AP-tagged form of TGF-α (AP-TGF-α) into parental HEK293 cells, the receptor was stimulated with either the most widely used GPR35 agonist zaprinast (39) (**Fig. 1*A***) or the recently described high-potency agonist lodoxamide (26) (Fig. 1*B*). These both promoted, in a concentration-dependent manner, cleavage of AP-TGF-α and its release from the surface of the cells. In HEK293 cells that had been genome-edited using clustered regularly interspaced short palindromic repeat (CRISPR)–Cas9 (CRISPR-associated protein-9) (30, 34, 35) to eliminate expression of all 4 of Gα_q_, Gα_11_, Gα_12_, and Gα_13,_ shedding of AP-TGF-α was no longer evident upon stimulation of hGPR35 (Fig. 1). Elimination of only a combination of Gα_12_ and Gα_13_ from HEK293 cells also prevented either of these hGPR35 active agonists from promoting AP-TGF-α cleavage (Fig. 1). By contrast, in a clone of HEK293 cells that had been genome-edited to eliminate expression of only Gα_q_ and Gα_11_ (34), the 2 phosphoinositidase C–linked G proteins that are expressed by these cells, and which, therefore, still expressed Gα_12_ and Gα_13_, both zaprinast and lodoxamide promoted shedding of AP-TGF-α as effectively, and with equal potency, as in the parental HEK293 cells (Fig. 1). In combination with the lack of effect of activation of hGPR35 in the Gα_12_/Gα_13_-null cells, this indicates that hGPR35 does not interact productively with Gα_q_/Gα_11_-family G proteins.

### Development and characterization of SPASM sensors

**Figure 2:**
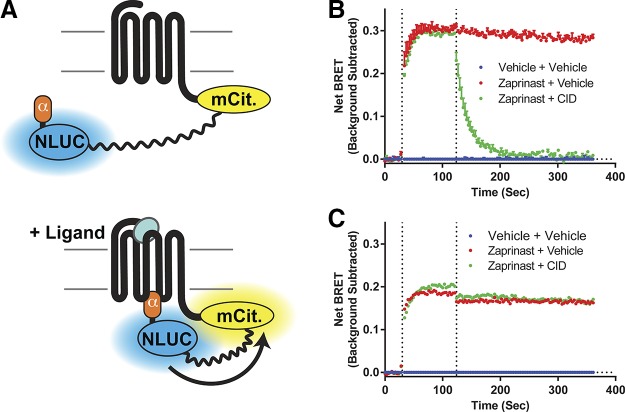
Kinetics of activation and deactivation of GPR35-Gα sensors. *A*) A diagram illustrates the nature of the GPR35–G protein sensors used and why G protein engagement induced by binding of an agonist ligand to the receptor is anticipated to result in enhanced BRET signal as the Nanoluc (NLUC) and mCitrine (mCit) components move closer together. *B*, *C*) Flp-In T-REx 293 cells stably harboring hGPR35-Gα_13_ (*B*) or mGPR35-Gα_13_ (*C*) were treated with doxycycline to induce construct expression. Kinetic studies were then performed to assess changes in BRET signal over time. Zaprinast (red) (EC_80_ 16 μM for human and 1 μM for mouse) or vehicle (blue) (HBSS with DMSO) were added at time 30 s (first vertical line) and maintained for a further 330 s. In certain studies, the human-specific GPR35 antagonist CID2745687 (CID) (green) (10 μM) was added at 120 s (second vertical line).

To provide improved quantification and to assess the relative degree of interaction of hGPR35 with Gα_12_ and Gα_13_ we generated a pair of GPCR– G protein SPASM sensors. In these the C-terminal tail of hGPR35 was linked in-frame to the C-terminal 27 aa of either G_12_ or G_13_ α subunits *via* a sequence that incorporated a BRET sensor that separates the luciferase Nanoluc from the fluorescent protein mCitrine with a 10 nm flexible linker (**Fig. 2*A***). Based on similar Fluorescence Resonance Energy Transfer–based SPASM sensors for other GPCRs (28, 40) and structural insights into the mechanisms of interactions between GPCRs and G proteins α subunits (7–12) we anticipated that following construct expression agonist-induced engagement of the G protein segment with the receptor would result in enhanced BRET signal. We cloned these sensors into Flp-In T-REx 293 cells and, following doxycycline-induced expression, studied initially the kinetics of response of the Gα_13_-containing sensor upon addition of zaprinast. Zaprinast produced a rapid increase in BRET that was maintained over a period of at least 6 min (Fig. 2*B*). CID2745687 has been described as a human ortholog specific antagonist of GPR35 (41, 42). Addition of CID2745687 after the zaprinast-induced BRET signal had reached a steady, maximal level rapidly reversed the effect of zaprinast at hGPR35-Gα_13_ (Fig. 2*B*). It is well appreciated that rodent orthologs of GPR35 display distinctly different ligand pharmacology than hGPR35 (20), but whether rodent forms of the receptor couple to the same G proteins as hGPR35 is uncertain. To assess this question, we generated equivalent mGPR35-Gα_12_ and -Gα_13_ sensors. Following similar stable expression in Flp-In T-REx 293 cells, zaprinast, which is also an effective agonist at mGPR35 (20), once again produced a large BRET signal at the Gα_13_-sensor that was maintained over time (Fig. 2*C*). Addition of CID2745687, however, failed to reverse the effect of zaprinast at mGPR35-Gα_13_ (Fig. 2*C*). This is consistent with previous *in vitro* studies showing that CID2745687 is a high-affinity antagonist at hGPR35 but has little affinity for mGPR35 (42). This suggests that reported effects of CID2745687 in cells and tissues from rodents (43, 44) probably reflect off-target effects of the compound rather than being mediated by GPR35.

**Figure 3:**
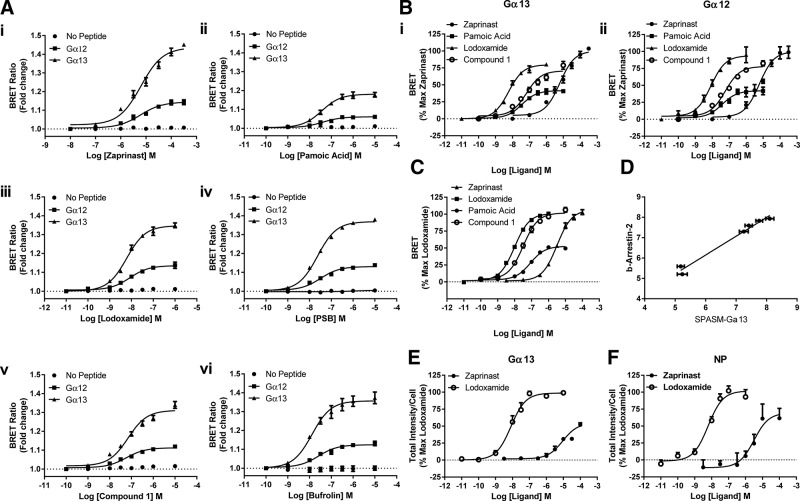
Validation and demonstration of effectiveness of hGPR35-Gα_12_ and hGPR35-Gα_13_ SPASM sensors. *A*) Flp-In T-REx 293 cells stably harboring hGPR35-Gα_13_ (squares), hGPR35-Gα_12_ (triangles), or hGPR35-NP (circles) were treated with doxycycline to induce construct expression; such cells were then treated with varying concentrations of zaprinast (*i*), pamoic acid (*ii*), lodoxamide (*iii*), PSB-13253 (PSB) (*iv*), compound 1 (*v*), or bufrolin (*vi*) for 5 min, and alteration in BRET signal was compared with basal. *B*) Data from *A* for the 4 denoted agonists are replotted, with maximal response to zaprinast defined as 100%. For both hGPR35-Gα_13_ (*i*) and hGPR35-Gα_12_ (*ii*), pamoic acid acted as a partial agonist compared with the other ligands. *C*) The same ligands as shown in *B* were used to assess interactions between hGPR35 and β-arrestin-2 in a BRET-based assay following transient cotransfection of hGPR35-eYFP and β-arrestin-2–*Renilla* luciferase into HEK293T cells. *D*) Correlation of potency of all 6 ligands shown in *A* in assays using the hGPR35-Gα_13_ sensor and the hGPR35a–β-arrestin-2 interaction assay is shown. *E*, *F*) Although the construction of the SPASM sensors adds a substantial molecular construct to the C-terminal tail of hGPR35, it does not prevent effective agonist-induced internalization of the constructs from the surface of Flp-In T-REx 293 cells expressing such constructs: hGPR35-Gα_13_ (*E*), hGPR35-NP (*F*).

A wide range of previously characterized ligands with agonism at hGPR35, including zaprinast (39) (**Fig. 3*A******i***), pamoic acid (41) (Fig. 3*A**ii*), lodoxamide (26) (Fig. 3*A**iii*), PSB-13253 (27) (Fig. 3*A**iv*), compound 1 (25) (Fig. 3*A**v*), and bufrolin (26) (Fig. 3*A**vi*), added to cells expressing hGPR35-Gα_13_ all produced robust and concentration-dependent increases in BRET signal. This was also the case for hGPR35-Gα_12_ (Fig. 3*A*). Although the measured EC_50_ of each of the agonists tested was very similar for activation of Gα_12_ and Gα_13_ (**Table 1**), in every case the maximal response of the Gα_13_-based sensor was markedly higher (Fig. 3*A*). To demonstrate the critical role of the G protein C-terminal sequence in promoting the observed increase in BRET signal, we also generated a control no-peptide (NP) hGPR35-SPASM construct that simply lacked such a C-terminal sequence. Following its stable expression and induction in Flp-In T-REx 293 cells, none of the agonists was able to enhance the basal BRET signal of the NP sensor (Fig. 3*A*). In further support of the ability of these hGPR35-SPASM sensors to report the pharmacological characteristics of GPR35 agonists appropriately, as illustrated in Fig. 3*B**i*, *ii*, pamoic acid, although potent, clearly acted as a partial agonist compared with the other ligands at both the Gα_13_- and Gα_12_-containing sensors. This is fully consistent with findings using other assay endpoints, including binding of [^35^S]GTPγS and receptor internalization (32). Of equal importance in further validating use of the SPASM sensors, the potency measures for activation of the Gα_13_-containing sensor by different agonists were highly correlated (*r*^2^ = 0.98) with values obtained in BRET-based hGPR35–β-arrestin-2 interaction assays (Fig. 3*C, D*) that have provided the most widely used approach to identify and characterize ligands at GPR35 (25, 26, 41, 42). Interaction with a β-arrestin is an important step in agonist-induced internalization of many GPCRs (34) including GPR35. The G protein–containing sensor constructs retained the capacity to be internalized from the surface of their host Flp-In T-REx 293 cells upon exposure to either the high-potency agonist lodoxamide or the lower-potency agonist zaprinast, as assessed using a high-content imaging platform that detects the intracellular location of the mCitrine fluorescent protein component of the sensors (Fig. 3*E*), and this was also the case for the equivalent hGPR35-NP sensor (Fig. 3*F*).

**TABLE 1:** Potency of ligands at hGPR35-Gα_12_ and hGPR35-Gα_13_ sensors

Ligand	hGPR35-Gα_12_ pEC_50_ ± sem	hGPR35-Gα_13_ pEC_50_ ± sem
Zaprinast	5.22 ± 0.10	5.20 ± 0.07
Pamoic acid	7.40 ± 0.15	7.44 ± 0.09
Lodoxamide	8.08 ± 0.09	8.22 ± 0.06
PSB-13253	7.49 ± 0.10	7.63 ± 0.04
Compound 1	7.33 ± 0.09	7.16 ± 0.08
Bufrolin	7.59 ± 0.11	7.89 ± 0.10

Data represent the mean ± sem from at least 3 independent experiments; p, negative logarithm.

### The molecular basis for selective coupling to Gα_13_

**Figure 4:**
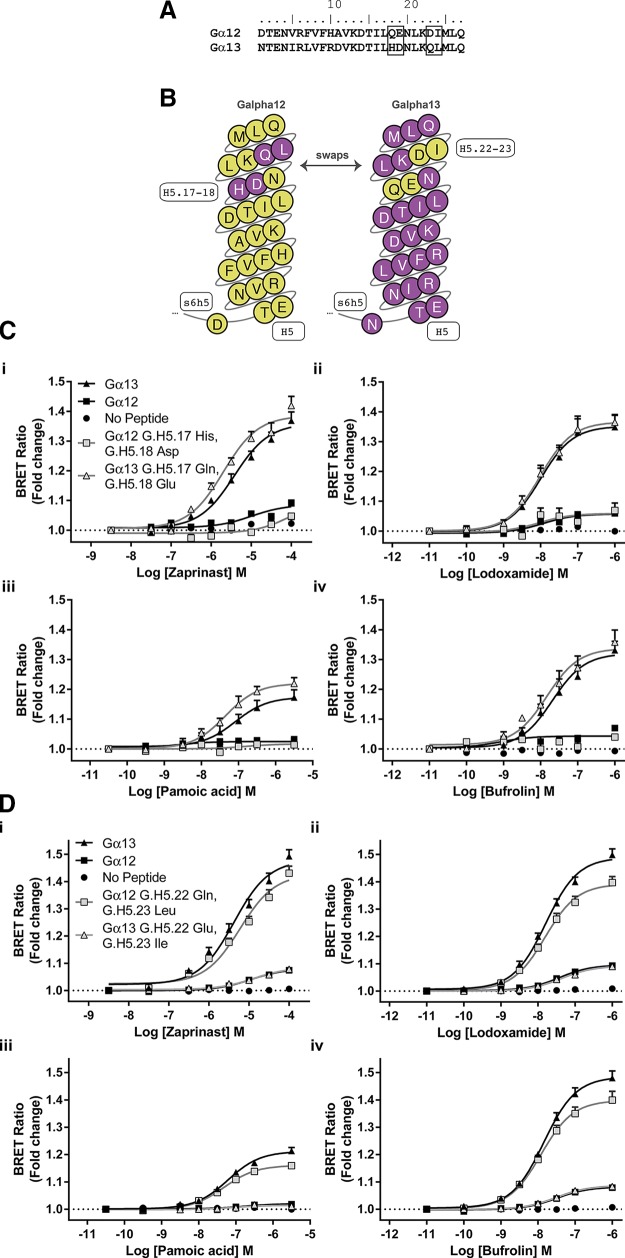
G_12_/G_13_ selectivity for hGPR35 resides within the C-terminal 10 aa. *A*) The C-terminal 27 aa of Gα_12_ and Gα_13_ are shown with amino acids that differ within the last 10 aa boxed. *B*) The Common Gα Numbering system (36, 37) is used to highlight these differences and their positions within the C-terminal G protein α5 helix. *C*, *D*) hGPR35-SPASM sensors were constructed in which residues at positions G.H5.17 and G.H5.18 (*C*) or G.H5.22 and G.H5.23 (*D*) were swapped between Gα_12_ and Gα_13_. Following stable expression and induction in Flp-In T-REx 293 cells, the ability of each of zaprinast (*i*), lodoxamide (*ii*), pamoic acid (*iii*), and bufrolin (*iv*) to enhance BRET signals was compared with the effect of these ligands at the hGPR35-Gα_13_, hGPR35-Gα_12_, and hGPR35-NP sensors.

The marked difference in effectiveness of agonists at the hGPR35-Gα_13_ sensor and the hGPR35-Gα_12_ sensor, coupled with earlier evidence that hGPR35 selectively interacts with Gα_13_ over Gα_12_ (32), led us to assess the molecular basis for this difference. Studies with chimeric G protein α subunits have shown that substitution of between 5 and 10 aa at the extreme C-terminal end of a G protein, which is within the α5 helix, is sufficient to switch GPCR selectivity (12). Within this region there are only 4 aa differences between Gα_13_ and Gα_12_ (**Fig. 4*A, B***). These differences are a pair of residues located at positions −10 [Common Gα Numbering system (35, 36) G.H5.17] and −9 (G.H5.18) and a pair of amino acids at positions −5 (G.H5.22) and −4 (G.H5.23) (Fig. 4*B*). Initially we swapped His and Asp at positions G.H5.17 (His) and G.H5.18 (Asp) of Gα_13_ for the equivalent amino acids, Gln and Glu, in Gα_12_ and *vice versa* in the context of the hGPR35 sensors. This had little effect on function compared with the wild type sequences (Fig. 4*C*), and this was the case for all hGPR35 agonists that we assessed (Fig. 4*C*). However, when we exchanged the residues Asp and Ile at positions G.H5.22 (Asp) and G.H5.23 (Ile) of Gα_12_ into Gα_13_ to replace Gln and Leu, activation by agonists resulted in as limited BRET signal as with full-length Gα_12_ (Fig. 4*D*). The opposite was true when Gln and Leu from Gα_13_ were used to replace Asp and Ile in the Gα_12_ sequence. This generated a sensor with substantial gain of function that was now almost equivalent to the full sequence from Gα_13_ (Fig. 4*D*). Once again, this was the case no matter whether zaprinast, lodoxamide, pamoic acid, or bufrolin was employed as the agonist (Fig. 4*D*).

**Figure 5:**
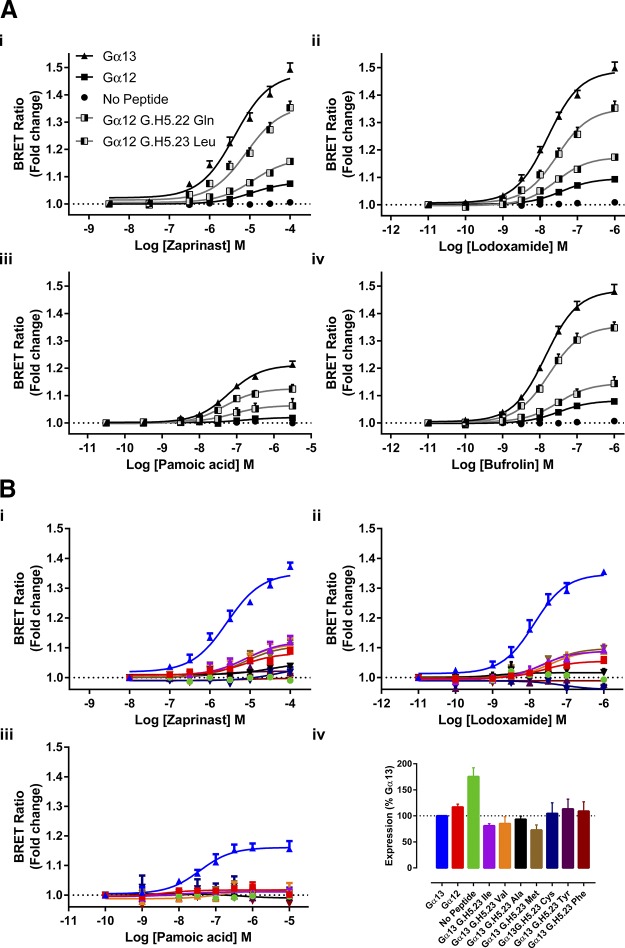
G_12_/G_13_ selectivity for hGPR35 is defined predominantly by a single leucine/isoleucine variation. *A*) In experiments akin to Fig. 4, hGPR35-G protein sensors were constructed and expressed in which single amino acids at positions G.H5.22 and G.H5.23 were swapped between Gα_12_ and Gα_13_, and the effect of the denoted ligands [zaprinast (*i*), lodoxamide (*ii*), pamoic acid (*iii*), and bufrolin (*iv*)] were compared with results generated using the hGPR35-Gα_13_, hGPR35-Gα_12_, and hGPR35-NP sensors. *B*) Position G.H5.23 (Leu) in Gα_13_ was altered to a number of other amino acids, and functionality was assessed in response to the noted GPR35 agonists [zaprinast (*i*), lodoxamide (*ii*), and pamoic acid (*iii*)], with parallel responses of hGPR35-Gα_13_, hGPR35-Gα_12_, and hGPR35-NP sensors recorded as controls. Direct measures of luciferase activity in cells transiently expressing these constructs defined their relative expression levels (*iv*).

We extended these studies by replacing only a single residue at a time. Remarkably, given the physiochemical similarities of Leu and Ile, substitution of Leu (G.H5.23) for Ile in Gα_12_ generated an hGPR35-SPASM sensor that responded almost as well to GPR35 agonists as the full sequence from Gα_13_ (**Fig. 5*A***). By contrast, substitution of Asp (G.H5.22) by Gln in Gα_12_, although it produced an enhanced response to each of zaprinast, lodoxamide, pamoic acid, and bufrolin compared with the wild type Gα_12_ sequence (Fig. 5*A*), resulted in a much more limited effect compared with the introduction of Leu (G.H5.23) in place of Ile. Finally, in this context we assessed whether Leu at position G.H5.23 was the only amino acid that could provide effective coupling between hGPR35 and a Gα_13_-based G protein. This was the case; alteration of this residue to any of Val, Ala, Met, Cys, Tyr, or Phe generated a sensor that was not activated more effectively than either G.H5.23 Ile Gα_13_ or full-length Gα_12_ (Fig. 5*B*), and once more this was true when assessing a range of chemically distinct GPR35 agonist ligands (Fig. 5*B*). These amino acids were selected because they cover the identity of residue G.H5.23 across mammalian G protein α subunits. Although BRET provides a ratiometric signal that is anticipated to be independent of construct expression level, we directly examined the relative expression levels of each of the G.H5.23 point mutant forms of the Gα_13_- and Gα_12_-containing GPR35 sensors used in these transient expression experiments by directly measuring luciferase activity (Fig. 5*B*). This examination showed that the Gα_12_ sensor construct was expressed at a very similar level as the Gα_13_ sensor construct, although it generated a very limited signal compared with the Gα_13_ sensor, and that alteration of residue G.H5.23 in the Gα_13_ sensor across the range of amino acids introduced had no substantial effect on construct expression levels. The GPR35-NP sensor was expressed at rather higher levels than the others, and this may indicate that the G protein segment of the other constructs reduces expression.

### This selectivity is maintained in full-length Gα_13_

**Figure 6:**
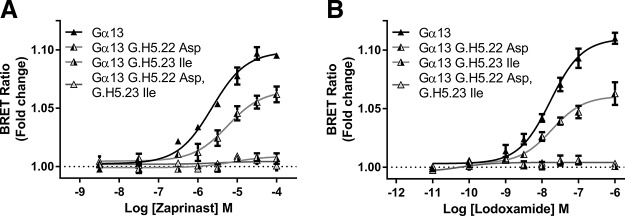
The importance of residue G.H5.23 in Gα_13_ sensors is maintained in the full-length G protein sequence. hGPR35-eYFP was coexpressed in HEK293 cells with forms of full-length Gα_13_ that either were wild type, had residue G.H5.22 converted to Asp, had residue G.H5.23 converted to Ile, or contained both of these alterations. Nanoluc had been introduced into all of the forms of Gα_13_ to provide a potential BRET pairing with the eYFP-tagged receptor. Cells were then stimulated with varying concentrations of either zaprinast (*A*) or lodoxamide (*B*). Data are shown as means ± sd (*n* = 3).

To ensure that outputs from the SPASM sensor studies would correlate with effects observed with the corresponding full-length G proteins, Gα_13_ was next modified by insertion of Nanoluc at residue position 128. This construct was cotransfected with hGPR35-eYFP into HEK293 cells lacking both Gα_12_ and Gα_13_, and BRET was measured as a surrogate of receptor-G protein interaction following addition of varying concentrations of zaprinast (**Fig. 6*A***) or lodoxamide (Fig. 6*B*). Subsequent studies employed this base G protein construct with residues G.H5.22 (Gln), G.H5.23 (Leu), or both altered to the corresponding residues of Gα_12_ (G.H5.22, Asp) or (G.H5.23, Ile) (Fig. 6). The results using these variants were very similar to those obtained using the equivalent SPASM sensors. The largest signal was achieved using wild type full-length Gα_13_ (Fig. 6). Replacement of G.H5.22 (Gln) by Asp reduced interactions between receptor and G protein to a small extent, whereas replacement of G.H5.23 (Leu) by Ile or a combination of replacement of G.H5.22 (Gln) and G.H5.23 (Leu) by Asp and Ile resulted in forms of full-length Gα_13_ that were unable to interact effectively with GPR35 in an agonist-dependent manner (Fig. 6). This was the case when either zaprinast (Fig. 6*A*) or lodoxamide (Fig. 6*B*) was used as agonist.

### Computational analysis

**Figure 7:**
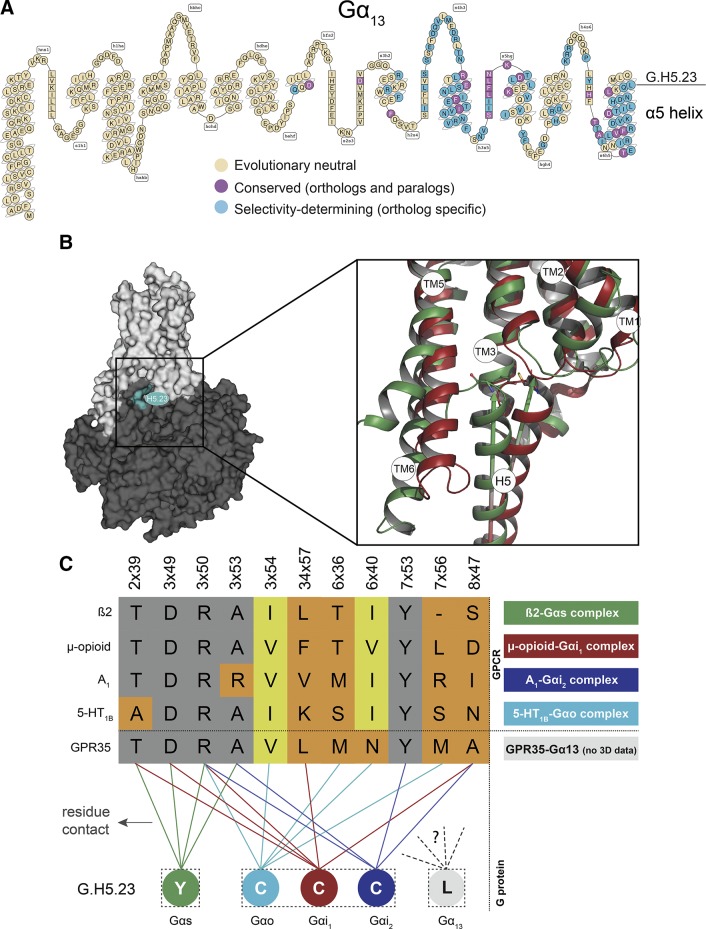
Modeling studies. *A*) Snake-like diagram of Gα_13_ with the G protein barcode highlighting evolutionary neutral, conserved, and selectivity-determining positions (barcode cutoff 0.96). *B*) Location of G.H5.23 (cyan) in the receptor-G protein complex (left) and comparison of the α5 helix domain C termini of Gα_s_ (green) and Gα_i1_ (red) (right). Arrows indicate the rotation differences between the α5 helix domains. Comparison of interface contacts and contacting residues between recently published GPCR–G protein structures are shown, as well as for the GPR35 G_12_/G_13_ selectivity-determining position G.H5.23. *C*) Alignment of G.H5.23 contacting receptor residue positions (gray: conserved; yellow: differing). This suggests a structurally yet-to-be-defined, alternative binding mode and contact profile for the G_12_/G_13_ family subtypes and their receptor coupling partners.

The G.H5.23 position is not conserved among the human Gα subtype paralogs (7 Gα subtypes with Cys, 2 with Ile, 4 with Tyr, 1 with Phe, and 1 with Leu) but is highly conserved among the Gα_12_ and Gα_13_ orthologs. This renders it part of the selectivity-determining G protein barcode (36) (**Fig. 7*A***). Residue positions in this barcode represent the determinants of GPCR–G protein selectivity, as deduced from evolutionary conservation (conserved in orthologs but not in paralogs) (36). Given the limited structural coverage of receptors and complexes to model a GPR35-Gα_13_ complex, we looked at the currently available receptor–G protein complexes of µ-opioid receptor–Gα_i1_ (45), β_2_-adrenoceptor–Gα_s_ (8), adenosine A_1_ receptor–Gα_i2_ (46), and 5-HT_1A_–Gα_o1_ (47) to attempt to rationalize the importance of G.H5.23 for G_13_ function. A comparison between these structures reveals differences in the position of transmembrane receptor helix VI and in the orientation between the G protein’s α5 helix domain (∼20° rotation) (Fig. 7*B*). For the β_2_-adrenoceptor–Gα_s_ complex, G.H5.23 (Tyr) has hydrophobic contacts with positions 2×39, 3×49, 3×50, and 3×53 of the receptor, whereas G.H5.23 (Cys) contacts 2×39, 3×49, 3×50, 34×57 (intracellular loop 2), and 8×47 in the µ-opioid–Gα_i1_; 3×50, 3×54, 6×36, 6×40, and 7×53 in 5-HT_1B_–Gα_o_; and 3×50, 3×53, 7×53, and 8×47 in adenosine A_1_–Gα_i2_ (Fig. 7*C*). The distinct binding patterns of the available structures suggest there must be a further distinct binding mode for GPR35-Gα_13_. Thus, it is plausible that this specific binding pose is highly constrained in space and hence only Leu has the correct balance of hydrophobicity, flexibility, isotropic surface area, and electrostatic properties.

### Introduction of G.H5.23 Leu into Gα_q_ allows coupling to GPR35

**Figure 8:**
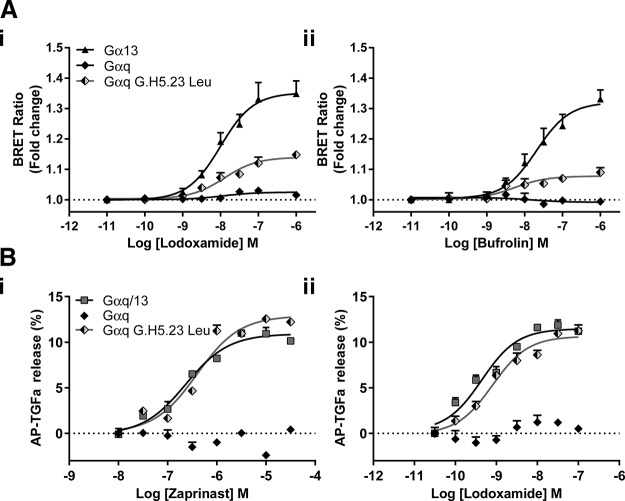
Replacement of Gα_q_ G.H5.23 Tyr by Leu allows engagement of Gα_q_ by agonist-occupied hGPR35. *A*) The ability of varying concentrations of either lodoxamide (*i*) or bufrolin (*ii*) to enhance BRET at hGPR35-SPASM sensors containing the C-terminal 27 aa of Gα_q_ (filled diamonds), G.H5.23Leu Gα_q_ (shaded diamonds), or Gα_13_ (triangles) was assessed. *B*) The ability of varying concentrations of either zaprinast (*i*) or lodoxamide (*ii*) to promote shedding of an AP-tagged form of TGF-α was assessed following cotransfection of hGPR35 and AP-TGF-α with either full-length Gα_q_ (solid diamonds), G.H5.23 Leu Gα_q_ (shaded diamonds), or chimeric Gα_q_/Gα_13_ containing the C-terminal hexapeptide of Gα_13_ (squares) into HEK293 cells that had been genome-edited to lack expression of a combination of Gα_q_, Gα_11_, Gα_12_, and Gα_13_. Basal levels of AP-TGF-α release were subtracted. Data represent means ± sd in triplicates from each group of a single experiment that is representative of 3 performed.

It is intriguing, therefore, that although a GPR35-Gα_q_ SPASM sensor was not activated by GPR35 agonists (**Fig. 8*A***), replacement of G.H5.23 (Tyr in Gα_q_) by Leu also generated a sensor for GPR35 that provided a substantial level of functionality (Fig. 8*A*). To also extend this to the context of full-length G protein α subunits, we generated a Tyr-Leu mutation at G.H5.23 in Gα_q_ and introduced this variant, along with hGPR35, into HEK293 cells genome-edited to lack each of Gα_q_, Gα_11_, Gα_12_, and Gα_13_. Now, in TGF-α shedding studies, both zaprinast and lodoxamide were able to promote release of AP-TGF-α in a concentration-dependent manner (Fig. 8*B*) and with potency for the 2 agonists that was not significantly different from that observed in either parental HEK293 or Gα_q_ plus Gα_11_ knock-out HEK293 cells (Fig. 1). Moreover, the effectiveness of TGF-α shedding induced by either of these GPR35 agonists when activating G.H5.23 Leu Gα_q_ was equivalent to that observed when we instead introduced into the genome-edited cells lacking each of Gα_q_, Gα_11_, Gα_12_, and Gα_13_ a chimeric form of Gα_q_/Gα_13_ in which all of the C-terminal 6 aa were derived from Gα_13_ (Fig. 8*B*).

## DISCUSSION

Although many GPCRs display a level of promiscuity in coupling to several families of heterotrimeric G proteins, there is little known about potential receptor selectivity between more closely related members of each of the 4 broad, generic families. Detailed studies on the G_s_, G_i_, and G_q_ families reflect their direct roles in the regulation of levels of secondary messengers and that toxins and chemical tools that disrupt signaling *via* members of these families are available and are used widely (12, 48). By contrast, the G_12_/G_13_ group is much less studied, although these play central roles in cytoskeletal organization and signaling *via* Rho-family small monomeric G proteins. It is also clear that many GPCRs can and do cause activation of these G proteins, but direct, easy-to-measure assays of their stimulation are not broadly available. Certain GPCRs do, however, appear to display considerable selectivity for G_12_/G_13_ over other G proteins, and in the case of the nominally orphan receptor GPR35 it has been suggested that there is even marked selectivity for G_13_ over G_12_ (49). To assess this in a controlled and potentially quantitative manner, we established a range of SPASM sensors (28, 40) in which hGPR35a was linked to the C-terminal 27 aa of various G protein α subunits *via* a flexible linker in which receptor interaction with the G protein segment results in enhanced BRET signal. A variant NP form, lacking the G protein segment, did not result in altered BRET with addition of various agonists at hGPR35, demonstrating that the G protein segment was indeed required for signal alteration. Moreover, in agreement with studies conducted using a TGF-α shedding assay performed in cells from which various G protein α subunits had been removed *via* genome editing, an hGPR35-Gα_q_ SPASM sensor did not respond to GPR35 agonist ligands.

A number of key outcomes were produced. First, using a sensor containing hGPR35 and the C-terminal 27 aa of Gα_13_, a large increase in BRET signal was generated following the addition of a range of chemically distinct agonists. This sensor provided an appropriate measure of ligand potency because the potency profile in this assay was highly correlated with outcomes from an hGPR35–β-arrestin-2 interaction assay that has been widely used to identify novel agonists at this receptor (26, 27, 41, 42). Moreover, it also provided a suitable estimate of agonist efficacy because pamoic acid clearly functioned as a partial agonist, as previously defined in a range of other assays. A further key observation that validated the use of the sensors was that although a sensor containing mGPR35 was also activated effectively by zaprinast, which has similar potency at mGPR35 and hGPR35 (20), CID2745687, which is a human-specific antagonist of GPR35 (20, 42), was unable to reverse the effect of zaprinast at the mGPR35-containing sensor. It is important to highlight that although hGPR35 and mGPR35 are poorly conserved overall in their sequences, they do engage effectively with the same G protein in Gα_13_. Secondly, although the agonists tested are able to also activate Gα_12_ in such a sensor construct, the maximal signal of all GPR35 agonists tested was substantially lower than for Gα_13_. Because this assay generates a ratiometric signal, variations in response would not be anticipated to be related to expression level. Moreover, direct measures of the fluorescence of the mCitrine component of the BRET sensor indicated very similar expression levels of hGPR35-Gα_13_ and hGPR35-Gα_12_. As such, these outcomes indicated either that hGPR35 interacts substantially less effectively with Gα_12_ than with Gα_13_ or that marked variations in the orientation of these interactions alter the proximity of the Nanoluc-mCitrine BRET pair within the sensor. Although it is impossible to separate these possibilities conclusively, the fact that alteration of a single amino acid (G.H5.23) between Leu and Ile produced signal-level switches close to those observed for the full sequences of Gα_13_ and Gα_12_ strongly favors the effect as being an intrinsic difference in interaction of the receptor between the 2 proteins. Moreover, replacement of Leu at position G.H5.23 in the sequence of Gα_13_ with any amino acid we tried resulted in a loss of agonist-induced BRET to a level at or below those obtained with the Gα_12_ sequence. A sensor containing the C-terminal 27 aa of Gα_q_/Gα_11_ (these 2 G proteins are identical over this region) showed no agonist-induced signal, but, remarkably, simple replacement of Tyr G.H5.23 by Leu generated a substantial level of interaction, and this was also observed in the TGF-α shedding assay when we introduced Leu in place of Tyr in full-length Gα_q_.

Although GPCR and cognate G protein structure determination is advancing rapidly (8, 45–47), there are little direct data on how much difference might be expected for a receptor in complex with 2 different G proteins. Van Eps *et al.* (50) have suggested differences in the ways in which 2 distinct GPCRs interact with their cognate G proteins, but the outcomes are predictions rather than direct comparisons. Kleinau *et al.* (51) have taken a mutagenic approach to address selective interactions of the thyrotropin receptor with G_s_ and with G_q_, but these studies focused on the receptor rather than the G protein. A study with a larger direct relevance used the SPASM approach also employed herein to assess differences in interactions between receptors with Gα_s_ and Gα_q_, 2 still markedly different G proteins from different families (40). Furthermore, an extensive computational analysis on the potential basis for GPCR–G protein selectivity by Flock *et al.* (36) explored a potential barcode for receptor selectivity within the amino acid sequences at the C-terminal region of different G proteins. However, this remains challenging to interpret fully in the absence of a broader range of atomic-level structures and provided no insights into potential selectivity between Gα_13_ and Gα_12_, perhaps in part because these are the G proteins for which the most limited GPCR interaction profiles have been reported.

We have also taken advantage of the most recent data on natural genetic variations in the human population from the Genome Aggregation Database, consisting of 123,136 exome sequences (52). Strikingly, no natural mutations have been observed in any individual at G.H5.23 in Gα_12_ or Gα_13_, which suggests that G.H5.23 in G_12_/G_13_ is under strong selection in the human population. Interestingly, a Gα_13_ G.H5.23 Leu374-Ile mutation and a Gα_12_ G.H5.23 Ile378-Ser mutation have been reported in the Catalogue of Somatic Mutations in Cancer (53). In the case of the Gα_13_ G.H5.23 Leu374-Ile mutation, our study suggests this is linked to reduced function in response to GPR35 activation, and it will be interesting to explore if this is also the case for other Gα_13_-interacting receptors. GPR35 has been reported to be up-regulated in breast cancer tissue compared with normal adjacent tissue (54), but the overall significance of this remains uncertain.

Taken together, Gα_13_ Leu374 (G.H5.23) seems to be the single most relevant residue for G protein selectivity, at least for GPR35. However, the molecular details of this phenomenon are yet to be elucidated in structural and mutational studies (on the receptor side) to pinpoint the G_12_/G_13_ specific receptor interface partners of G.H5.23.

## Abbreviations

AP: alkaline phosphatase
BRET: bioluminescence resonance energy transfer
CID2745687: GPR35 antagonist
eYFP: enhanced yellow fluorescent protein
GPR35: G protein–coupled receptor 35
HEK293: human embryonic kidney 293
hGPR35: human GPR35
mGPR35: mouse GPR35
NP: no peptide
PSB-13253: 6-bromo-8-(4-methoxybenzamido)-4-oxo-4H-chromene-2-carboxylic acid
SPASM: systematic protein affinity strength modulation
